# Selective UMLS knowledge infusion for biomedical question answering

**DOI:** 10.1038/s41598-023-41423-8

**Published:** 2023-08-30

**Authors:** Hyeryun Park, Jiye Son, Jeongwon Min, Jinwook Choi

**Affiliations:** 1 Interdisciplinary Program for Bioengineering, Seoul National University Graduate School, Seoul, Republic of Korea; 2 Integrated Major in Innovative Medical Science, Seoul National University Graduate School, Seoul, Republic of Korea; 3 Department of Biomedical Engineering, College of Medicine, Seoul National University, 103, Daehak-ro, Jongno-gu, Seoul, Republic of Korea

**Keywords:** Health services, Computational models

## Abstract

One of the artificial intelligence applications in the biomedical field is knowledge-intensive question-answering. As domain expertise is particularly crucial in this field, we propose a method for efficiently infusing biomedical knowledge into pretrained language models, ultimately targeting biomedical question-answering. Transferring all semantics of a large knowledge graph into the entire model requires too many parameters, increasing computational cost and time. We investigate an efficient approach that leverages adapters to inject Unified Medical Language System knowledge into pretrained language models, and we question the need to use all semantics in the knowledge graph. This study focuses on strategies of partitioning knowledge graph and either discarding or merging some for more efficient pretraining. According to the results of three biomedical question answering finetuning datasets, the adapters pretrained on semantically partitioned group showed more efficient performance in terms of evaluation metrics, required parameters, and time. The results also show that discarding groups with fewer concepts is a better direction for small datasets, and merging these groups is better for large dataset. Furthermore, the metric results show a slight improvement, demonstrating that the adapter methodology is rather insensitive to the group formulation.

## Introduction

As the use of artificial intelligence increases in all fields, many application systems are being introduced in the medical field. One of the applications in medicine is question-answering (QA) for doctors seeking clinical evidence of a diagnosis or treatment, or for the general public finding information about their health conditions^1^. QA is a task that requires not only an understanding of the context, but also knowledge of the subject. In particular, biomedical QA requires accuracy and expertise as it is closely related to patient safety issues^2^. Biomedical QA research using knowledge bases has been developed, but there is still room for improvement^1^. In this study, we introduce a method to infuse the Unified Medical Language System (UMLS) knowledge more efficiently into pretrained language models for biomedical QA and discuss its effects.

Pretrained language models should fully utilize their acquired contextual information to handle knowledge-intensive tasks, such as QA, fact-checking, and dialogue tasks^3^. The biomedical domain, like any other domain, requires relevant knowledge to solve problems. In order to answer biomedical questions, it is important to understand the relation between concepts such as “hypoventilation (concept), cause of (relation), respiratory acidosis (concept)”. Pretrained language models learn information contextually using a self-attention mechanism, but they do not utilize knowledge contexts such as entity semantics or relationships between entities^4^. Recent studies have shown that models trained with masked language modeling have difficulty capturing rich factual knowledge^5^. BERT is overly reliant on the surface form of entity names^6^ and mostly did not learn the meaning of negation^7^. As our target is biomedical QA, the language model should not just rely on the surface form of biomedical concepts or relations.

Knowledge bases are useful for extracting semantic knowledge by recognizing nodes as concepts and edges as relations^8^. Leveraging knowledge bases notably improves performance for knowledge-intensive tasks^4^. Knowledge bases in the general domain include ConceptNet^9^, WordNet^10^, and the atlas of machine commonsense^11^. The UMLS is one of the well-known knowledge bases in the biomedical domain^12^.

Several studies have examined knowledge infusion into large pretrained language models, such as the BERT^13^, RoBERTa^14^, and others. Most models are jointly pretrained with masked language modeling and knowledge infusion objectives^15–21^. As pretraining is expensive in terms of computation cost and time, several studies have only fine-tuned their models with structural modifications, such as incorporating a knowledge layer^22^ or using a selective attention mechanism^4^. Another approach, parameter efficient pretraining and fine-tuning can leverage multiple adapters to inject various types of knowledge. The K-Adapter model^5^ is a RoBERTa with two adapters: a factual adapter pretrained with a relation classification task and a linguistic adapter pretrained with dependency relation prediction. The mixture-of-partition (MoP) method^23^ partitions the UMLS knowledge base into subgraphs, and adapters connected to a biomedical BERT are pretrained for each group. The pretrained adapters can then be integrated for fine-tuning.

**Figure 1:**
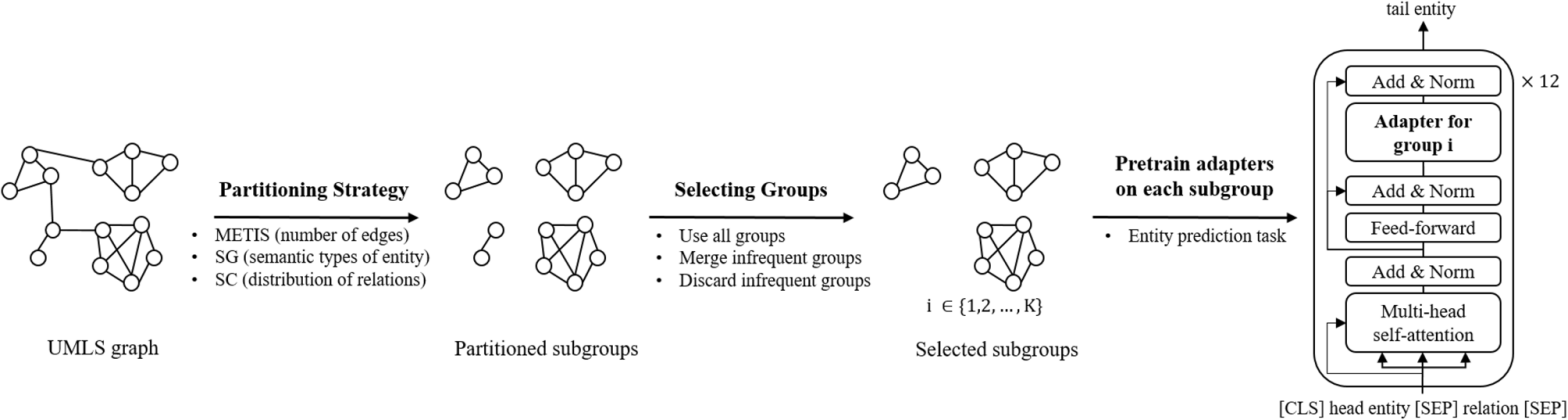
Overview of partitioning, selecting groups, and pretraining adapters. The strategies of partitioning the UMLS knowledge graph and selecting groups produce K sub-groups. SG indicates semantic groups, SC denotes semantic-type collections, and the partitioning criteria are in parentheses. The knowledge of each group is injected into each adapter by an entity prediction task.

This study investigates the need to use all semantics in a knowledge graph when injecting knowledge into adapters. Our work is an extension of the MoP approach but differs in strategies of grouping large UMLS knowledge base, and selecting subgroups for more efficient pretraining, as shown in Fig. 1. The original MoP uses the METIS software package^24^ to divide the knowledge graph based on its number of edges between vertices, resulting in groups of similar size. In contrast, we utilized semantic groups (SG) and semantic-type collections (SC) to organize groups according to the relationship of semantic types. As group sizes are disproportionate, we experimented with methods of selecting subgroups. In three biomedical QA datasets which are BioASQ7b^25^, PubMedQA^26^, and MedQA^27^, adapters pretrained on semantically partitioned groups showed more efficient performance. For small finetuning datasets such as BioASQ7b, PubMedQA, and sampled MedQA, it was better to discard groups with a small number of concept unique identifiers (CUI), while for large dataset like MedQA, it was better to merge these groups. Since the QA datasets for a specific domain are relatively small, removing groups with fewer concepts seems to be an efficient way without significantly affecting the metric scores. In the case of general domain, where QA datasets and knowledge graphs are larger, more research is needed on merging groups with fewer concepts rather than discarding them, in order to achieve efficient training. In addition, automatically partitioning METIS algorithm and semantically partitioning and merging or discarding groups with fewer concepts show similar performance without statistically significant difference. This rather demonstrates that the adapter methodology is rather insensitive to the group formulation. In other words, when injecting knowledge from UMLS into adapters, how the graph is grouped has little effect on the metric, but it reduces the computational parameters and time. As most of the groups with fewer concepts are less relevant to the QA datasets, merging or removing them reduces the number of groups the model has to reference.

## Methods

### Overall training scheme

**Figure 2:**
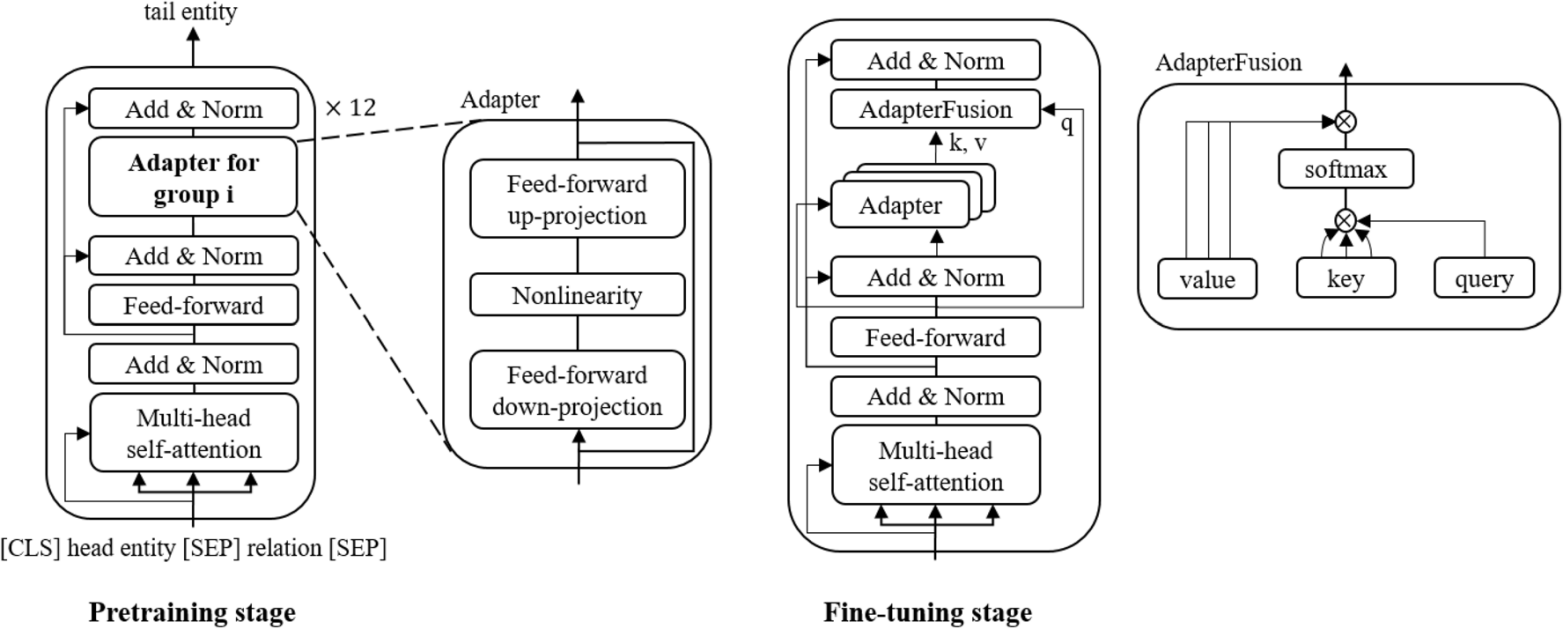
Overview of pretraining and finetuning stage. The pretraining task is predicting the tail entity, given a head entity and a relation as input. During pretraining, PubMedBERT weights are fixed, only adapter and prediction head weights are learned. All parameters are updated in the QA fine-tuning phase, and given question and passage as input, the model predicts an answer (yes/no/maybe). While finetuning, AdapterFusion component integrates adapters with knowledge of subgroups.

Transformer adapters^28,29^ are one of the lightweight finetuning methods that require training with only small number of model parameters. The PubMedBERT^30^ model used has 12 transformer layers and each transformer layer has an additional adapter part as shown in Fig. 2 pretraining stage. Transformer adapters can have various customization options, such as the placement of learnable weights, residual connections, and bottleneck sizes^31^.

Our models are based on a pretrained PubMedBERT^30^ with multiple adapters, each adapter following Pfeiffer configuration ^31^ as shown in Fig. 2. The adapter down-projects features into a smaller dimension, applies a nonlinearity, and then up-projects to the original dimension. To inject knowledge, each adapter is pretrained on each partitioned subgroup using an entity prediction task; a tail entity is predicted, given a head entity and a relation. During pretraining, PubMedBERT weights are frozen, and only the adapter and prediction head weights are learned with the entity prediction task. The knowledge injected adapters are then used for finetuning QA task. While finetuning, the AdapterFusion^29^ integrates adapters by activating adapters related to question and given passage. As shown in Fig. 2, the query vector is the output of the feed-forward layer and the key and value vectors are the output of the adapters. Similar to the attention mechanism, if the query and key vector are similar, the dot product of those vectors will be higher, resulting in a higher attention score. The attention score is a weight of the value vector, with higher weight indicating more active adapter. In this study, we investigate the need to use whole UMLS knowledge graph and the group formulation methods for more efficient knowledge infusion.

### Baseline method

The UMLS triplets contain a head entity, relation, and tail entity. To generate the triplets, obtain concept unique identifier (CUI) and concept string (STR) from MRCONSO.RRF file, and get relation (RELA) between two CUIs from MRREL.RRF file. The baseline MoP approach^23^, which achieved the best performance at that time, partitions the UMLS knowledge graph of the SNOMED CT (US Edition 2020AA) by using METIS. The METIS algorithm considers the number of edges between vertices and does not regard relations. Three phases are applied: coarsening, initial partitioning, and un-coarsening. We reimplemented the MoP in our experimental settings to obtain two versions of partitioned groups: Sfull-METIS-20 and S20Rel-METIS-20. Sfull-METIS-20 uses all 229 relations while S20Rel-METIS-20 uses only the top-20 most frequent relations. As the previous work^23^ tested with knowledge graph of 5, 10, 20, 40, and 60 partitioned groups, the knowledge graph with 20 groups showed the best performance, we set 20 groups as a default setting for both experiments.

### Semantically partitioning method

The UMLS provides a semantic network^32^ consisting of 133 semantic types which are broad categories of entities and 54 semantic relations. Since the 133 semantic types are still complex to comprehend the knowledge, several studies have attempted to create smaller, coarser groups^33–36^. We utilized two semantically partitioned groups of UMLS: semantic groups (SG)^36^ and semantic-type collections (SC)^34^. SG is the result of grouping the semantic types into 15 higher-level categories. The six grouping principles of SG are semantic validity (semantic coherency within the groups), parsimony (minimize the number of groups), completeness (encompass the entire domain), exclusivity (each concept must be in only one group), naturalness (acceptable to domain experts), and utility (useful for a specific purpose)^36^. There are total of 28 groups in SC, grouping semantic types with exactly the same set of relations, in other words, grouping semantic types that are structurally identical and semantically close^34^.

**Figure 3:**
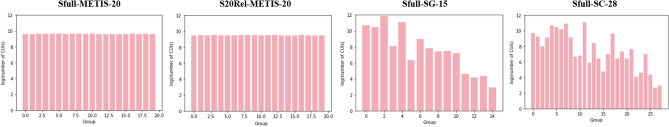
A log scale distribution of the number of CUIs per group according to different partitioning strategies (METIS, SG, and SC).

As Sfull consists of triplets (entity 1-relation-entity 2) generated from UMLS, we had to match the entities to SG and SC groups. First, the CUI for each entity was obtained from the UMLS MRCONSO.RRF file, the Type Unique Identifier (TUI) for each CUI was matched from the Semantic Network’s MRSTY.RRF file, and the full semantic type name of the TUI was acquired from the SRDEF file. As a result, all of the following information was matched to the triplet: entity 1, relation, entity 2, CUI 1 (CUI for entity 1), CUI 2 (CUI for entity 2), TUI 1 (TUI for CUI 1), TUI 2 (TUI for CUI 2). Then, the SG group name was matched to the TUI using the UMLS SemGroups.txt file. For each SC, we manually matched the SC group name with corresponding TUI by referring to the paper^34^. The Fig. 3 illustrates the distribution of the number of CUIs in each group. Using the METIS algorithm, the 20 groups have a relatively uniform distribution on CUIs, while due to the semantic partitioning, SG and SC exhibit large imbalances in the number of CUIs among the groups.

Thus, we experimented with merging or discarding subgroups, as the semantically partitioned group size was highly imbalanced. In SG, the minimum number of CUIs within a group is 18, the maximum is 140,058, and the average is 20,155. In SC, the minimum number of CUIs in a group is 15, the maximum is 63,842, and the average is 10,797. The infrequent group in this paper is a group with fewer CUIs than the threshold. This threshold is set in two cases. First, when conducting experiments to train the adapter with various numbers of data, 1000 is commonly used as the smallest number^37,38^, so the threshold is set to 1000 and groups with less than 1000 CUIs are either merged or removed. In the second case, the threshold is the average number of CUIs among the groups, with SG set to 20,155 and SC set to 10,797. For SG, infrequent groups are simply merged or removed based on the number of CUIs in the group. Sfull-SG-15 uses all 15 groups, Sfull-SG-11 merges groups with less than 1000 CUIs, and Sfull-SG-10 removes those groups. Sfull-SG-5 merges groups with CUIs less than the average number of CUIs (20,155), and Sfull-SG-4 eliminates these groups. For SC, infrequent groups are either deleted or merged with their parent groups according to the SC hierarchy in the paper^34^. Sfull-SC-28 uses all 28 groups, Sfull-SC-20 and Sfull-SC-16 handle groups with less than 1000 CUIs, whereas Sfull-SC-12 and Sfull-SC-7 handle those with CUIs fewer than the mean (10,797). The Supplementary Fig. S1 shows the percentage of CUIs used for each knowledge graph. Sfull-METIS-20, Sfull-SG-15, and Sfull-SC-28 all have a total of 302,332 CUIs, and the merging cases of infrequent groups, Sfull-SG-11, Sfull-SG-5, Sfull-SC-20, and Sfull-SC-12 also have 302,332 CUIs. For cases where infrequent groups are discarded, Sfull-SG-10 has 301,517 CUIs (99.73%), Sfull-SG-4 contains 282,913 CUIs (93.58%), Sfull-SC-16 includes 297,996 CUIs (98.56%), and Sfull-SC-7 has 255,085 CUIs (84.37%).

After partitioning and selecting groups, the next step is pretraining adapters. To inject knowledge, each adapter is pretrained on each partitioned subgroup through an entity prediction task using triplets; given a head entity (CUI) and a relation, a tail entity (CUI) is predicted. During pretraining, PubMedBERT weights are frozen, and only the adapter and prediction head weights are learned. The knowledge injected adapters are then used for finetuning QA task.

### Finetuning stage

After injecting knowledge into adapters, the whole model is fine-tuned on the biomedical QA datasets by updating all model parameters including adapters, PubMedBERT, and the prediction head. The BioASQ7b dataset has four types of questions: factoid, yes/no, summary, and list. As in a previous work^23^, we used only 885 yes/no questions such as “Is Baloxavir effective for influenza?”. Each question and sentences from PubMed abstract is annotated with the answer (yes/no). The PubMedQA-labeled dataset has 1000 instances of questions generated from article titles, abstracts excluding conclusions, and yes/no/maybe answers. As the BioASQ7b and PubMedQA datasets are small, we ran the finetuning experiment ten times and averaged the results. The MedQA dataset has 12,723 questions, with multiple-choice answers generated from professional medical board exams. Table 1 lists the details of the datasets.

**Table 1:** Statistical details of the three biomedical QA datasets.

Dataset	Total	Train/dev/test	Yes/no(/maybe)
BioASQ7b	885	670/75/140	80%/20%
PubMedQA	1000	450/50/500	55.2%/33.8%/11.0%
MedQA	12,723	10,178/1272/1273	X

There are several differences in finetuning between the original MoP and the reimplementation MoP. Due to the class imbalance in the QA datasets, f1 score is more reliable than accuracy. While the original MoP only tested based on accuracy alone, we also evaluated using the macro-precision, macro-recall, and macro-f1 score. After finetuning, the model of the best training step should be used for evaluation. The original MoP used accuracy as a criterion, but for this experiment we selected the model with the highest f1 score. The statistical comparisions between original and reimplementation MoP are shown in Supplementary Table S1. For the BioASQ7b dataset, our reimplemented accuracy was statistically higher than the original accuracy. For the PubMedQA dataset, there was no statistically significant difference between our reimplemented accuracy and the original accuracy.

### Experimental settings

All experiments were performed using two 3090 RTX GPUs. Whereas the original MoP pretrained the adapters for one or two epochs with a random seed, we pretrained our model for ten epochs and used a fixed seed of 42, resulting in slightly different results. During pretraining, the learning rate was 1e−4, batch size was 256, and AdamW optimizer was used with 0.01 weight decay. During fine-tuning, the learning rate was 1e−5, batch size was eight, total epoch was 25, and model used early stopping with a patience of five. The only difference with MedQA fine-tuning was that the dataset size was large; thus, the batch size was set to two. In addition, BioASQ7b and PubMedQA experiments were repeated ten times with a seed list consisting of ten fixed seeds (42, 64, 128, 256, 512, 1024, 2048, 4096, 8192, 16,384) while original MoP used a seed list with ten random seeds. As MedQA is a large dataset we only experimented once with a fixed seed 42 for reproducibility. The pretraining objective was cross-entropy loss for the entity prediction task and the fine-tuning objective was cross-entropy loss for the biomedical QA task.

## Results

### Metric evaluation

As BioASQ7b and PubMedQA provide yes/no and yes/no/maybe classification tasks, respectively, the evaluation metrics include accuracy, macro-precision, macro-recall, and macro-f1 scores. Additionally, because both the datasets are imbalanced, the macro-f1 score is more reliable. For MedQA, accuracy is used for multiple-choice classification. In contrast from the previous study, when adapters were pretrained for ten epochs, the Sfull knowledge graph showed higher scores than S20Rel in all datasets. In the BioASQ7b and PubMedQA datasets, performance often improved when adapters were pretrained with the semantic group instead of the METIS group, and the required parameters and time were reduced. With the MedQA dataset, when using the semantic group, efficient training was accomplished with the use of reduced parameters and in a timely manner than the METIS group, but the performance was comparable.

Table 2 shows the fine-tuning results of the BioASQ7b dataset, for which the format of the adapter name uses graph-partitioning method-number of groups. If only PubMedBERT is finetuned, the macro-f1 score is 0.8493, 0.8719 when using the adapter without knowledge infusion, and 0.8921 when using the adapters pretrained with the entire METIS group. The best SG-pretrained adapter was Sfull-SG-4, which performed slightly better than Sfull-METIS-20 while reducing the parameters and learning times. The best adapter pretrained with SC was Sfull-SC-12, which had a lower macro-f1 score than the Sfull-METIS-20, but reduced the number of parameters and time required. In most cases, removing groups based on the number of CUIs in each group was better than merging.

**Table 2:** Results show that the best adapter is Sfull-SG-4, which is pretrained on Unified Medical Language System semantic groups and discarded infrequent groups.

Adapter status	Adapter name	Parameters	Time	Accuracy	Macro-precision	Macro-recall	Macro-f1
X	X	109,483,778	45 m	0.8750 ± 0.0012	0.8794 ± 0.0011	0.8370 ± 0.0031	0.8493 ± 0.0023
Not pretrained	X	110,378,306	50 m	0.8921 ± 0.0012	0.8911 ± 0.0010	0.8603 ± 0.0024	0.8719 ± 0.0020
Pretrained with METIS group	S20Rel-METIS-20	166,332,674	2 h11 m	0.9071^**†**^ ± 0.0002	0.9100^**†**^ ± 0.0007	0.8798^**†**^± 0.0003	0.8913^**†**^ ± 0.0003
	Sfull-METIS-20	166,332,674	2 h17 m	0.9093^**†**^ ± 0.0003	0.9171^**†**^ ± 0.0002	0.8775 ± 0.0008	0.8921^**†**^ ± 0.0005
Pretrained with UMLS semantic groups	Sfull-SG-15 (use all groups)	157,433,473	1 h42 m	0.8964 ± 0.0011	0.8940 ± 0.0011	0.8702 ± 0.0024	0.8782 ± 0.0020
	Sfull-SG-11 (merge groups under 1000)	150,314,114	1 h39 m	0.8929 ± 0.0013	0.8982 ± 0.0020	0.8575 ± 0.0021	0.8723 ± 0.0020
	Sfull-SG-10 (remove groups under 1000)	148,534,274	1 h24 m	0.8979 ± 0.0029	0.8952 ± 0.0042	0.8745 ± 0.0040	0.8815 ± 0.0040
	Sfull-SG-5 (merge groups under 20,155)	139,635,074	1h22m	0.9007 ± 0.0005	0.9020 ± 0.0006	0.8706 ± 0.0008	0.8830 ± 0.0007
	Sfull-SG-4 (remove groups under 20,155)	137,855,234	1 h13 m	0.9093^**†**^ ± 0.0004	0.9165^**†**^ ± 0.0004	0.8780 ± 0.0010	0.8922^**†**^ ± 0.0007
Pretrained with semantic-type collections	Sfull-SC-28 (use all groups)	180,571,394	2 h38 m	0.8886 ± 0.0029	0.8822 ± 0.0037	0.8626 ± 0.0050	0.8691 ± 0.0047
	Sfull-SC-20 (merge groups under 1000)	166,332,674	2 h3 m	0.8957 ± 0.0016	0.9018 ± 0.0014	0.8602 ± 0.0034	0.8742 ± 0.0028
	Sfull-SC-16 (remove groups under 1000)	159,213,314	2 h2 m	0.9007 ± 0.0023	0.9035 ± 0.0020	0.8706 ± 0.0049	0.8806 ± 0.0042
	Sfull-SC-12 (merge groups under 10,797)	152,093,954	1 h35 m	0.9057^**†**^ ± 0.0002	0.9013 ± 0.0005	0.8865^**†**^ ± 0.0004	0.8914^**†**^ ± 0.0003
	Sfull-SC-7 (remove groups under 10,797)	143,194,754	1 h22 m	0.9022 ± 0.0006	0.9078 ± 0.0007	0.8689 ± 0.0011	0.8837 ± 0.0009

The Sfull-SG-4 adapter exhibits similar performance to the Sfull-METIS-20, and reduces parameters and computation time. ^**†**^Indicates a significant difference between PubMedBERT with knowledge infused adapters and PubMedBERT without adapters (independent t-test, p < 0.05).

Table 3 shows the results of using the PubMedQA dataset. According to the macro-f1 score, using SG and SC was superior to using METIS, but there was no statistically significant difference. Finetuning the PubMedBERT alone results in macro-f1 score of 0.4336, 0.4394 when using the adapter without knowledge injection, and 0.4402 using the adapters pretrained with the full METIS group. The best adapters were Sfull-SG-10 and Sfull-SC-16, which discarded groups with less than 1000 CUIs. Similarly, for groups under the CUI mean frequency, the elimination method performed better than merging. For PubMedQA, utilizing semantic groups resulted in higher macro-f1 scores and fewer parameters and training times.

**Table 3:** For the PubMedQA dataset, adapters pretrained with SG and SC are both superior to the METIS group in terms of macro-f1 score, required parameters, and time.

Adapter status	Adapter name	Parameters	Time	Accuracy	Macro-precision	Macro-recall	Macro-f1
X	X	109,483,778	40 m	0.5918 ± 0.0026	0.4597 ± 0.0037	0.4413 ± 0.0028	0.4336 ± 0.0035
Not pretrained	X	110,379,075	42 m	0.6094 ± 0.0017	0.4628 ± 0.0021	0.4506 ± 0.0019	0.4394 ± 0.0019
Pretrained with METIS group	S20Rel-METIS-20	166,333,443	1 h10 m	0.5938 ± 0.0027	0.4509 ± 0.0044	0.4364 ± 0.0024	0.4293 ± 0.0029
	Sfull-METIS-20	166,333,443	1 h41 m	0.5994 ± 0.0035	0.4519 ± 0.0058	0.4484 ± 0.0034	0.4402 ± 0.0043
Pretrained with UMLS semantic groups	Sfull-SG-15 (use all groups)	157,434,243	1 h27 m	0.6042 ± 0.0033	0.4714 ± 0.0087	0.4516 ± 0.0019	0.4489 ± 0.0019
	Sfull-SG-11 (merge groups under 1000)	150,314,883	1 h5 m	0.5966 ± 0.0017	0.4533 ± 0.0054	0.4528 ± 0.0027	0.4417 ± 0.0041
	Sfull-SG-10 (remove groups under 1000)	148,535,043	1 h15 m	0.6098^**†**^ ± 0.0007	0.4668 ± 0.0020	0.4561 ± 0.0012	0.4501 ± 0.0019
	Sfull-SG-5 (merge groups under 20,155)	139,635,843	1 h4 m	0.5518 ± 0.0021	0.4386 ± 0.0021	0.4294 ± 0.0015	0.4144 ± 0.0017
	Sfull-SG-4 (remove groups under 20,155)	137,856,003	1 h9 m	0.5980 ± 0.0012	0.4679 ± 0.0027	0.4492 ± 0.0014	0.4473 ± 0.0016
Pretrained with semantic-type collections	Sfull-SC-28 (use all groups)	180,572,163	1 h59 m	0.5810 ± 0.0027	0.4909 ± 0.0094	0.4373 ± 0.0018	0.4305 ± 0.0022
	Sfull-SC-20 (merge groups under 1000)	166,333,443	1 h50 m	0.6028 ± 0.0023	0.4918 ± 0.0079	0.4525 ± 0.0022	0.4468 ± 0.0027
	Sfull-SC-16 (remove groups under 1000)	159,214,083	1 h33 m	0.6000 ± 0.0022	0.4669 ± 0.0001	0.4524 ± 0.0014	0.4480 ± 0.0013
	Sfull-SC-12 (merge groups under 10,797)	152,094,723	1 h17 m	0.5794 ± 0.0015	0.4391 ± 0.0042	0.4299 ± 0.0031	0.4214 ± 0.0039
	Sfull-SC-7 (remove groups under 10,797)	143,195,523	1 h6 m	0.5886 ± 0.0019	0.4785 ± 0.0034	0.4467 ± 0.0023	0.4389 ± 0.0033

Both Sfull-SG-10 and Sfull-SC-16 adapters eliminate the infrequent groups rather than merging them. † has the same meaning as in Table 2.

As the results of BioASQ7b and PubMedQA showed that SG shows better performance than SC, only SG was used with the MedQA dataset; Table 4 shows the obtained results. Using the adapter significantly improved performance compared with only finetuning PubMedBERT and using adapter without knowledge infusion. The accuracies of Sfull-METIS-20 and Sfull-SG-5 adapters tied for the best score but using SG significantly reduced parameters and time. Differing slightly from the trend of other datasets, the infrequent-group merge method outperformed the discard method. As MedQA has more data than either BioASQ7b or PubMedQA, which means there are more questions that require more diverse knowledge, it might be better to incorporate all the knowledge, even though the meanings may be mixed. To support this, we further experimented with 509 instances randomly sampled from the MedQA training data, as there are 670 samples in BioASQ and 450 instances in PubMedQA. The results show that the best adapter was Sfull-SG-4, removing infrequent groups, as shown in Table 5. This indicates that for small datasets it is better to remove infrequent groups, and for large datasets it is better to merge them. Furthermore, for the metrics in most datasets, our model of semantically partitioned group shows a slightly better improvement over the METIS group, but there is no statistically significant difference. Comparing to the PubMedBERT without adapters, Sfull-METIS-20, S20Rel-METIS-20, SFull-SG-4, and SFull-SC-12 have a statistically significant difference in the BioASQ7b, and only Sfull-SG-10 has a statistically significant difference in the PubMedQA. This result asserts that the injection of UMLS into adapters, even though the semantic grouping has little effect on the metric, but it does show much benefits in terms of the computational parameters and time.

**Table 4:** For the large MedQA, Sfull-METIS-20 and Sfull-SG-5 adapters show the same accuracy but using SG significantly reduces parameters and time, allowing for efficient fine-tuning.

Adapter status	Adapter name	Parameters	Time	Accuracy
X	X	109,483,778	3 h2 m	0.3386
Not pretrained	X	110,377,537	4 h48 m	0.3425
Pretrained with METIS group	S20Rel-METIS-20	166,331,905	15 h50 m	0.3747
	Sfull-METIS-20	166,331,905	29 h23 m	0.3849
Pretrained with UMLS semantic groups	Sfull-SG-15 (use all groups)	157,432,705	14 h58 m	0.3778
	Sfull-SG-11 (merge groups under 1000)	150,313,345	9 h44 m	0.3778
	Sfull-SG-10 (remove groups under 1000)	148,533,505	10 h5 m	0.3747
	Sfull-SG-5 (merge groups under 20,155)	139,634,305	13 h42 m	0.3849
	Sfull-SG-4 (remove groups under 20,155)	137,854,465	9 h23 m	0.3621

**Table 5:** For the small sampled MedQA, the best method is to remove the infrequent groups, which show the same trend as the other small datasets, BioASQ and PubMedQA.

Adapter status	Adapter name	Accuracy
Pretrained with METIS group	Sfull-METIS-20	0.2663
Pretrained with UMLS semantic groups	Sfull-SG-15 (use all groups)	0.2828
	Sfull-SG-11 (merge groups under 1000)	0.2844
	Sfull-SG-10 (remove groups under 1000)	0.2671
	Sfull-SG-5 (merge groups under 20,155)	0.2820
	Sfull-SG-4 (remove groups under 20,155)	0.2899

## Discussion

### Impact of semantic group adapters

Since the partitioned groups are based on semantics, we experimented the performance of each adapter to examine the contribution of each group. The Supplementary Fig. S2 shows the macro-f1 score of each adapter from Sfull-SG-4, which performed best on the BioASQ7b test dataset, and Sfull-SG-10, which achieved the highest performance on the PubMedQA test dataset. The Sfull-SG-4 has four adapters: chemicals and drugs, anatomy, disorders, and procedures. The chemicals and drugs group includes entities related to chemical (protein, enzyme, etc.), clinical drug, and pharmacologic substance. The anatomy group contains anatomical structure (body, organ, tissue, cell, etc.) and body substance (extracellular material). The disorders group encompasses entities of abnormality, disease, symptom, finding, and dysfunction. In the procedures group, there are entities related to procedures, methods or techniques for diagnosis, examination, treatment, genetic research, etc., research activities, and healthcare activities such as patient care. As shown in the Supplementary Fig. S2, the procedures group contributes the most and the disorders group the least. Since most yes/no questions in BioASQ7b does not ask about one entity, but rather asks about how a specific treatment affects a disease, the procedures semantic group that includes procedures, methods, techniques, and research contents may have the greatest impact. In the case of PubMedQA, the physiology group contributes the most and the disorder group the least. The physiology group includes the physiologic function of cell, gene, molecule, organism, organ, and tissue, clinical and organismal attribute, and mental process. For Sfull-SG-10, the removed groups are geographic areas group, occupations group, organizations group, gene and molecular sequences group, and activities and behaviors group. Since most of these groups are less related to BioASQ7b and PubMedQA, removing them will yield more efficient training. However, it is a concern to remove the gene and molecular sequences group based on the number of concepts, so further research is necessary to augment groups using other knowledge graphs such as GenomicKB^39^.

To figure out which adapters contribute more depending on the input question, there are some examples in the Supplementary Table S2. The attention weight of the adapter fusion is the score of which adapter is concentrated. Question 1 is about the effect of Semagacestat, a candidate drug for Alzheimer's disease, and the question 2 asks about Axitinib, a small molecule tyrosine kinase inhibitor for pancreatic cancer, so it can be seen that the weight of the clinicals and drugs adapter and disorder adapter is relatively higher than other questions. As question 3 asks about the exosomal marker, which is an extracellular material in the body and Question 4 is about the activator of pancreatic stellate cells, both have a higher weight for the anatomy adapter than the other questions. Question 5 asks about the association of Miller-Dieker syndrome and abnormalities of chromosome 1, and question 6 asks about a complication of sinusitis, so the attention weight of the disorder group with information on abnormalities and diseases is higher. Lastly, looking at the questions with a high attention weights on the procedures group, question 7 is about gene therapy for auditory function, and question 8 asks about FDA approval that is activity. Therefore, it can be observed that each question requires different knowledge, and to answer the question the knowledge from appropriate semantic group adapters should be integrated through the different attention weights.

### Case study (incorrect answers)

For 140 BioASQ test dataset, when all groups were used without merging or discarding, all models using Sfull-METIS, Sfull-SG-15, and Sfull-SC-28 were wrong in three cases. Negative expressions were not recognized or different expressions with the same meaning, such as "not available” and "did not come yet” were not recognized as equivalent. In three cases where only Sfull-SG-15 was wrong, the given passage for the question was very long, so it is possible that the model did not catch the relevant part. Also, the answer to question “Tocilizumab is an anti-TNF antibody, yes or no?” is “no”, but the model seems to predict “yes” as the two concepts frequently appear together in the passage. The Sfull-SC-28 was wrong in two cases. The answer to the question “Does lucatumumab bind to CD140?” is “no”, but the passage says that lucatumumab binds to CD40, leading to model prediction of “yes” which may indicate the weakness at understanding numbers. The Sfull-METIS-20 was wrong in five cases, and there were cases where the answer was incorrect even if the answer was clearly in the passage. Also, if the question mentions Gepotidacin, but the passage has a different name, GSK2140944, the model does not recognize that the two mean the same thing.

In summary, when using the METIS group, there are cases where the prediction is wrong even if there is a clear answer in the passage, but semantically grouped SG or SC do not have such case. The weakness of all models is that they are vulnerable to negative expressions or different expressions of the same meaning. If there are synonyms for medical terms, the model can be supplemented by adding those to the knowledge graph.

### Limitations

When training with a small amount of data, such as BioASQ7b, PubMedQA, and sampled MedQA, merging infrequent groups can mix meanings and confuse the model. On the other hand, discarding infrequent groups may prevent the model from answering questions related to those groups. Differing slightly from the trend of other datasets, for the data-heavy MedQA, merging was better way even when the semantics were mixed. Although the discarding method is a better direction for medical applications because the datasets in this field are most often small, more research is needed on large-scale biomedical QA datasets like MedQA.

The discrepancy between knowledge injection pretraining task (entity prediction) and finetuning task (yes/no/maybe or multiple-choice classification) makes it difficult to deeply understand how the model is predicting. For explanatory power, using BioASQ factoid or list questions whose answers are entities, or generating the BioASQ's ideal answer (summaries of relevant snippets) can be further research directions. If a model finds an answer in a given text or generates an answer, we can infer why the model made wrong predictions, but simply classifying yes/no/maybe leads to weak explanatory power. As adapters can be used for any transformer-based models, future researches can proceed beyond classification tasks to understand model predictions.

## Conclusion

Pre-trained language models can benefit from knowledge-infused adapters and this study questioned the need to use whole UMLS knowledge graph and the group formulation methods. We compared partitioning strategies, where METIS focused on the number of edges, whereas SG and SC considered the semantic type and distribution of relations, respectively. Using semantically partitioned groups to pretrain adapters showed more efficient performance than METIS groups in terms of the evaluation metrics, required parameters, and time. The method of discarding infrequent groups was preferable to merging in small finetuning datasets: BioASQ7b, PubMedQA, and small sampled MedQA. Conversely, for the data-rich MedQA, merging was better way even when the semantics were mixed. Although more research is needed on large-scale biomedical QA methods, the discarding method is a better direction for medical applications because the finetuning datasets in this field are most often small. In summary, to efficiently inject large knowledge graphs into adapters, it is not necessary to use the entire knowledge graph and the way of group formulation has little effect on the metric scores, but it does affect the computational parameters and time. Adapters can be used for any transformer-based models; thus, future research can improve other QA types, such as finding answers in documents and generating answers. Furthermore, more research on selecting the adapters with relevant knowledge could be another direction to enhance the model.

### Supplementary Information

Supplementary Information.

## Data Availability

The three datasets used in this study are already publicly available. We plan to upload the adapters pretrained on semantically partitioned UMLS knowledge to AdapterHub for easy use.
