# Supplementary material for: Selective UMLS knowledge infusion for biomedical question answering
Source: Sci Rep. 2023 Aug 30;13:14214. doi: 10.1038/s41598-023-41423-8 (PMC10468517; doi:10.1038/s41598-023-41423-8)
Supplement: Supplementary file 1 — Supplementary Information. [file 41598_2023_41423_MOESM1_ESM.pdf]

Manuscript Information

Title: Selective UMLS knowledge infusion for biomedical question answering

Submission ID: 006ac01f-3fb3-4f49-aa48-4afde17f9a68

Authors

Hyeryun Park[1,2], Jiye Son[1,2], Jeongwon Min[1,2], and Jinwook Choi[2,3,*]

[1]Interdisciplinary Program for Bioengineering, Seoul National University Graduate School

[2]Integrated Major in Innovative Medical Science, Seoul National University Graduate School

[3]Department of Biomedical Engineering, College of Medicine, Seoul National University

SUPPLEMENTARY INFORMATION

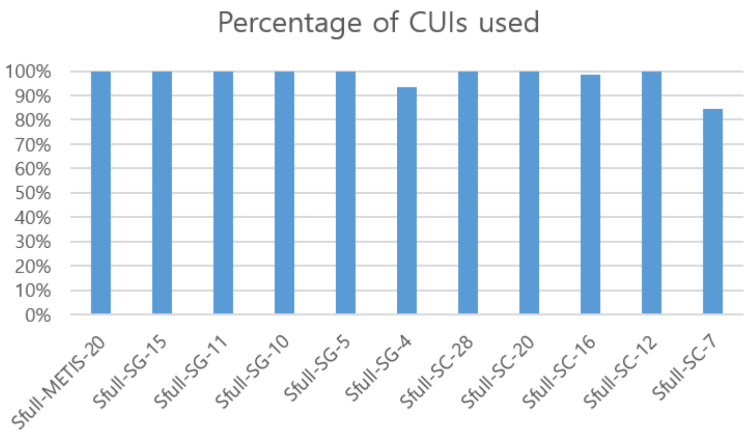

Supplementary Figure S1: The percentage of CUIs across all groups used for each knowledge graph.

Supplementary Table S1: Statistical comparisons between the original and reimplemented MoP method in terms of accuracy for three QA datasets. For the BioASQ7b dataset, † indicates that our reimplemented accuracies are statistically higher than the original accuracies(1 sample t-test, $p < 0.05$). For the PubMedQA dataset, there is no statistically significant difference between our reimplemented accuracy and the original accuracy.

**Adapter status**	**Adapter name**	**Datasets**	**Accuracy**	**Macro-precision**	**Macro-recall**	**Macro-f1**
Pretrained with METIS group (**original**)	S20Rel-METIS-20	BioASQ7b	0.9064	·	·	·
	Sfull-METIS-20	BioASQ7b	0.8864	·	·	·
Pretrained with METIS group (**reimplementation**)	S20Rel-METIS-20	BioASQ7b	**0.9071**† ±0.0002	0.9100 ±0.0007	0.8798 ±0.0003	0.8913 ±0.0003
	Sfull-METIS-20	BioASQ7b	**0.9093**† ±0.0003	0.9171 ±0.0002	0.8775 ±0.0008	0.8921 ±0.0005
Pretrained with METIS group (**original**)	S20Rel-METIS-20	PubMedQA	0.6284	·	·	·
	Sfull-METIS-20	PubMedQA	0.6174	·	·	·
Pretrained with METIS group (**reimplementation**)	S20Rel-METIS-20	PubMedQA	0.5938 ±0.0027	0.4509 ±0.0044	0.4364 ±0.0024	0.4293 ±0.0029
	Sfull-METIS-20	PubMedQA	0.5994 ±0.0035	0.4519 ±0.0058	0.4484 ±0.0034	0.4402 ±0.0043
Pretrained with METIS group (**original**)	S20Rel-METIS-20	MedQA	0.3802	·	·	·
	Sfull-METIS-20	MedQA	0.3633	·	·	·
Pretrained with METIS group (**reimplementation**)	S20Rel-METIS-20	MedQA	0.3747	·	·	·
	Sfull-METIS-20	MedQA	0.3849	·	·	·

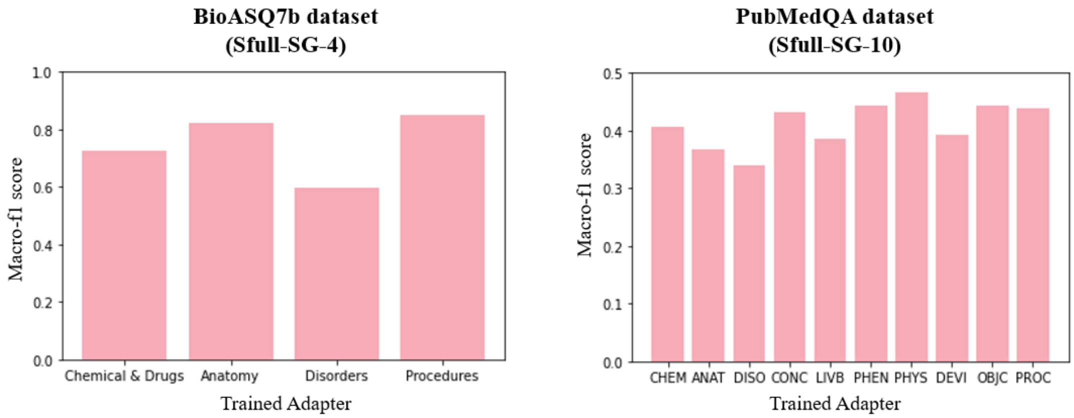

Supplementary Figure S2: The macro-f1 score of each adapter from Sfull-SG-4 for the BioASQ7b dataset and Sfull-SG-10 for the PubMedQA dataset. For Sfull-SG-10 groups, CHEM stands for chemical & drugs, ANAT is anatomy, DISO is disorders, CONC is concepts & ideas, LIVB is Living Beings, PHEN is phenomena, PHYS is physiology, DEVI is devices, OBJC is Objects, and PROC indicates procedures.

Supplementary Table S2: The attention weight of the Sfull-SG-4 adapter fusion shows which adapters are concentrated on for each input question. The Sfull-SG-4 consists of four adapters: chemicals & drugs, anatomy, disorders, and procedures.

Question	Chemicals & Drugs	Anatomy	Disorders	Procedures
1. Is Semagacestat effective for treatment of Alzheimer's disease?	**0.1980**	0.2827	**0.1224**	0.3968
2. Does Axitinib prolong survival of Pancreatic Cancer patients?	**0.1994**	0.2746	**0.1315**	0.3946
3. Is CD63 an exosomal marker?	0.1482	**0.2975**	0.0996	0.4547
4. Is TNF-α an activator of pancreatic stellate cells?	0.1523	**0.2918**	0.1011	0.4549
5. Is Miller-Dieker syndrome associated with abnormalities of chromosome 1?	0.1690	0.2783	**0.1314**	0.4113
6. Is subdural empyema a complication of sinusitis?	0.1547	0.2761	**0.1248**	0.4440
7. Can gene therapy restore auditory function?	0.1535	0.2819	0.1034	**0.4612**
8. Has ivosidenib been FDA approved for use against acute myeloid leukemia?	0.1530	0.2867	0.0957	**0.4646**
